# Association between economic development level and tuberculosis registered incidence in Shandong, China

**DOI:** 10.1186/s12889-020-09627-z

**Published:** 2020-10-16

**Authors:** Qian-yun Zhang, Dong-mei Yang, Lin-qing Cao, Jin-yue Liu, Ning-ning Tao, Yi-fan Li, Yao Liu, Wan-mei Song, Ting-ting Xu, Shi-jin Li, Qi-qi An, Si-qi Liu, Lei Gao, Wan-yan Song, Huai-chen Li

**Affiliations:** 1 Department of Respiratory Medicine, Shandong Provincial Hospital Affiliated to Shandong University, Shandong Provincial Hospital Affiliated to Shandong First Medical University, No. 324, Jingwuweiqi Road, Huaiyin District, Jinan, 250021 Shandong China; 2 Cheeloo College of Medicine, Shandong University, Jinan, 250012 Shandong China; 3 College of statistics, Shandong University of Finance and Economics, Jinan, 250202 Shandong China; 4 Department of Intensive Care Unit, Shandong Provincial Third Hospital, Jinan, 100191 Shandong China; 5 Peking Union Medical College, Beijing, 100005 China; 6 NHC Key Laboratory of Systems Biology of Pathogens, Institute of Pathogen Biology, and Center for Tuberculosis Research, Chinese Academy of Medical Sciences and Peking Union Medical College, 100730 Beijing, P. R. China; 7 International College, Jiangxi University of Finance and Economics, Nanchang, Jiangxi China; 8 Shandong University of Traditional Chinese Medicine, Jinan, 250355 Shandong China

**Keywords:** Tuberculosis, Registered incidence, Economic development

## Abstract

**Background:**

Tuberculosis (TB) is one of the major infectious diseases that seriously endanger people’s health. In Shandong province, the relationship between the level of economic development and TB incidence has not been studied. This study aims to provide more research basis for the government to prevent and control TB by exploring the impact of different economic factors on TB incidence.

**Methods:**

By constructing threshold regression model (TRM), we described the extent to which different economic factors contribute to TB registered incidence and differences in TB registered incidence among seventeen cities with different levels of economic development in Shandong province, China, during 2006–2017. Data were retrieved from the China Information System for Disease Control and Prevention.

**Results:**

Per capita medical expenditure (regression coefficient, -0.0314462; SD, 0.0079305; P > |t|, 0.000) and per capita savings (regression coefficient, 0.0001924; SD, 0.0000566; P > |t|, 0.001) passed the significance test at the level of 1%.They are the two economic indicators that have the greatest impact on TB registered incidence. Through the threshold test, we selected the per capita savings as the threshold variable. In the three stages of per capita savings (<9772.8086 China Yuan(CNY); 9772.8086–33,835.5391 CNY; >33,835.5391 CNY), rural per capita income always has a significant negative impact on the TB registered incidence (The regression coefficients are − 0.0015682, − 0.0028132 and − 0.0022253 respectively. P is 0.007,0.000 and 0.000 respectively.).In cities with good economies, TB registered incidence was 38.30% in 2006 and dropped to 25.10% by 2017. In cities with moderate economies, TB registered incidence peaked in 2008 at 43.10% and dropped to 27.1% by 2017.In poorer cities, TB registered incidence peaked in 2008 at 56.30% and dropped to 28.9% in 2017.

**Conclusion:**

We found that per capita savings and per capita medical expenditure are most closely related to the TB incidence. Therefore, relevant departments should formulate a more complete medical system and medical insurance policy to effectively solve the problem of “difficult and expensive medical treatment”. In order to further reduce the TB incidence, in addition to timely and accurate diagnosis and treatment, it is more important for governments to increase investment in medicine and health care.

## Background

Although the target of the Millennium Development Goals to halve tuberculosis prevalence and mortality by 2015, compared to 1990 levels, has been achieved around the world, TB remains a major infectious disease that threatens global health [1].The World Health Organization (WHO) estimates that 10 million people worldwide suffer from TB and 1.6 million people die in 2017.The social and economic impacts are devastating [2, 3].The End TB Strategy which was adopted by the World Health Assembly in May, 2014, outlines an overall target of reducing global TB incidence and mortality by 90 and 95% respectively by the year of 2035 [4] .This strategy emphasizes the importance of social determinants for prevention and care of TB, including policies to alleviate poverty, and social protection programme. The non-spatial ecological regression analyses conducted by Guy Harling and Marcia C. Castro found higher TB incidence associated with urbanization, population density, poor economic conditions, household crowding, worse health and healthcare indicators [5]. The content of this strategy is supported by many research results [6, 7]. Hence the 2010 lancet tuberculosis series appeal to a renewed emphasis on tackling poverty and promoting social protection to curb epidemics [8].At present, China is still one of the world’s 30 countries with a high burden of TB, with about 900,000 new cases of TB every year, ranking the third in the world [1]. In China, people either seek medical treatment voluntarily because of illness or routine medical examinations before employment and enrollment, and are diagnosed with tuberculosis through various laboratory tests and imaging. Once the patient is diagnosed with tuberculosis, it will be immediately reported to the Center for Disease Control and Prevention (CDC) at all levels. Patients can get anti-tuberculosis drugs for free at designated hospitals, but the cost of their examinations is not free. And we found in the medical activities that most of the tuberculosis patients in outpatient clinics came from rural areas. Therefore, we want to study whether there is a relationship between the economy and the incidence of TB and what this relationship is like. Despite a lot of research on TB abroad, only a few studies have investigated the relationship between the level of economic development and the incidence of TB, especially in China [9–11]. It can be seen from foreign studies that increasing social protection and public health expenditure is closely related to reducing the incidence, mortality, and cure success rate of TB, but whether the results of foreign studies are applicable to China or Shandong Province is not yet known. Foreign countries mainly explore the impact of factors such as per capita GDP and population density on TB, as well as the differences in TB incidence among countries with different income levels [10, 12], and our research has refined economic factors based on their researches and increased per capita Indexes such as per capita savings, number of doctors per capita, number of beds per capita, and Engel’s coefficient, etc., to explore whether these factors will affect the incidence of TB. This study aims to examine the association between the registered incidence of TB and the level of economic development in Shandong Province with the data from seventeen cities in Shandong province from 2006 to 2017, and to find out which indicators in economic aspects contribute to TB prevention and control and to what extent, so as to provide a basis for the prevention and control of tuberculosis in Shandong Province.

## Methods

### Study population and setting

Short-course treatment and free drug delivery have led to a significant reduction in TB mortality. However, there are still many problems and challenges in TB control in China because of its huge population. The number of TB cases is still high. China’s current TB control service system and control capacity cannot meet the needs of the new situation. According to Shandong provincial bureau of statistics in 2017, Shandong’s GDP (gross domestic product) is 7.26 trillion yuan, accounting for 8.8% of the country’s total, ranking third in the country, with a total population of 10.06 million and 7.2% of the country’s total, ranking second in the country [13].Therefore, reducing the TB incidence in Shandong Province is of great significance to the prevention and control of tuberculosis in China. There are a total of 17 cities in Shandong Province, and we have included all rural and urban populations of these 17 cities into the scope of the study.

### Data sources and study design

The annual incidence of TB is one of the most important indicators in TB burden, which can reflect the public health significance of TB. We obtained data at the city level for TB registered incidence per 100,000 population from the Tuberculosis Information Management System, Chinese Center for Disease Control and Prevention (CDC). Once the hospital confirms the patient as a tuberculosis patient, it will be reported to the disease control center of each region within the prescribed time. The system of the CDC contains information about all tuberculosis patients in Shandong Province. In the panel data, we take the total registered incidence of urban and rural areas in each city every year as the TB registered incidence in that city. Misdiagnosis and missed diagnosis of tuberculosis, incomplete coverage of surveillance are the main reasons for uncertainty about published estimates. In clinical work, misdiagnosis and missed diagnosis of diseases are inevitable. Misdiagnosis or missed diagnosis will inevitably lead to high or low registered incidence of tuberculosis. However, the data provided by the CDC is the only official data, and we believe they have minimized the error. And data on population and economy were provided by Shandong Statistical Yearbook annually. Economic indicators included per capita GDP, urban per capita disposable income and rural annual net income, population density, urbanization rate, per capita medical expenditure and the per capita savings, number of beds per capita, number of doctors per capita, rural engel coefficient, urban engel coefficient. In the panel data, values of all economic indicators in this study are the average of the year.The data used in this paper are panel data, covering the years 2006–17 from seventeen cities in Shandong Province.

### Procedures

We consider the fixed effect model due to the different situations of each city. The fixed effect model is constructed as follows: *PTR*_*it*_ *= α*_*i*_ *+ β*_*1*_*GDP*_*it*_ *+ β*_*2*_*TI*_*it*_ *+ β*_*3*_*RI*_*it*_ *+ β*_*4*_*DOP*_*it*_ *+ β*_*5*_*UR*_*it*_ *+ β*_*6*_*ME*_*it*_ *+ β*_*7*_*HS*_*it*_ *+ β*_*8*_*BN*_*it*_ *+ β*_*9*_*DN*_*it*_ *+ β*_*10*_*REC*_*it*_ *+ β*_*11*_*TEC*_*it*_ *+ ε*_*it.*_ Wherein, *α*_*i*_ is the fixed effect of individuals, representing the special effect of the city *i* and reflecting the individual differences of each city. *β* is the coefficient of various variables.*ε*_*it*_ represents the random disturbance term. In order to further test the advantages of the fixed effect model, the Hausmann test was carried out. The Hausmann test results showed that the *P* value was 0.0051, rejecting the null hypothesis. Therefore, compared with the mixed model and the random effect model, the fixed effect model was the best.

The original tuberculosis registered incidence and economic indicator data we obtained are from 2005 to 2017.However, due to the large missing data in 2005, the data for this year were not included in the scope of our study. In order to ensure the comprehensiveness and availability of the collected data, we finally select the panel data of seventeen cities in Shandong province from 2006 to 2017.Due to some missing observations, interpolation method was used to supplement the data in the actual analysis process, and consumer price index (CPI) was used for the flattening of all economic indicators to make the data more representative. The interpolation method is that an interpolation line is established based on the previous data and the next data of the missing value in the column, and then the missing value is filled in by the function value of the missing point in the linear interpolation function. CPI refers to the consumer price index. It is an important data used by the government to measure inflation and an index that measures the overall level of prices in the market. It reflects the supply-demand relationship and price trends of consumer goods. Flattening variables is that we calculate the ratio of the CPI in 2007 to the CPI in 2006, and then divide the corresponding economic data by this ratio to get the final data, and so on for other years. By using CPI to flatten the data, the impact of price changes on the values of economic indicators can be eliminated so that the data is more representative. In this paper, the economics software STATA 14.0 is used for regression, and the regression results are shown in Table 1. In Table 1, the regression coefficient ± indicates that the economic indicator is positively or negatively correlated with the registered incidence rate, and the value represents the degree of increase or decrease.

**Table 1:** Regression Results of Economic Indicators

Variable	RC	SD	t	P > |t|
Per capita GDP	0.0001319	0.0000768	1.72	0.088*
Urban per capita disposable income	0.0003896	0.0001897	2.05	0.041**
Rural annual net income	-0.0017531	0.0007222	−2.43	0.016**
Population density	0.0983965	0.0492288	2.00	0.047**
Urbanization rate	−0.0544054	0.0990188	−0.55	0.583
Per capita medical expenditure	−0.0314462	0.0079305	−3.97	0.000***
Per capita savings	0.0001924	0.0000566	3.40	0.001***
Number of beds per capita	0.0005271	0.0014147	0.37	0.710
Number of doctors per capita	−0.0002612	0.0026471	−0.10	0.921
Rural engel coefficient	−0.161335	0.2303641	−0.70	0.485
Urban engel coefficient	0.1908406	0.0991439	1.92	0.056*

Note. *RC* Regression coefficient, *SD* Standard deviation, *GDP* Gross domestic product

The number of * indicates the strength of the correlation

**Fig. 1:**
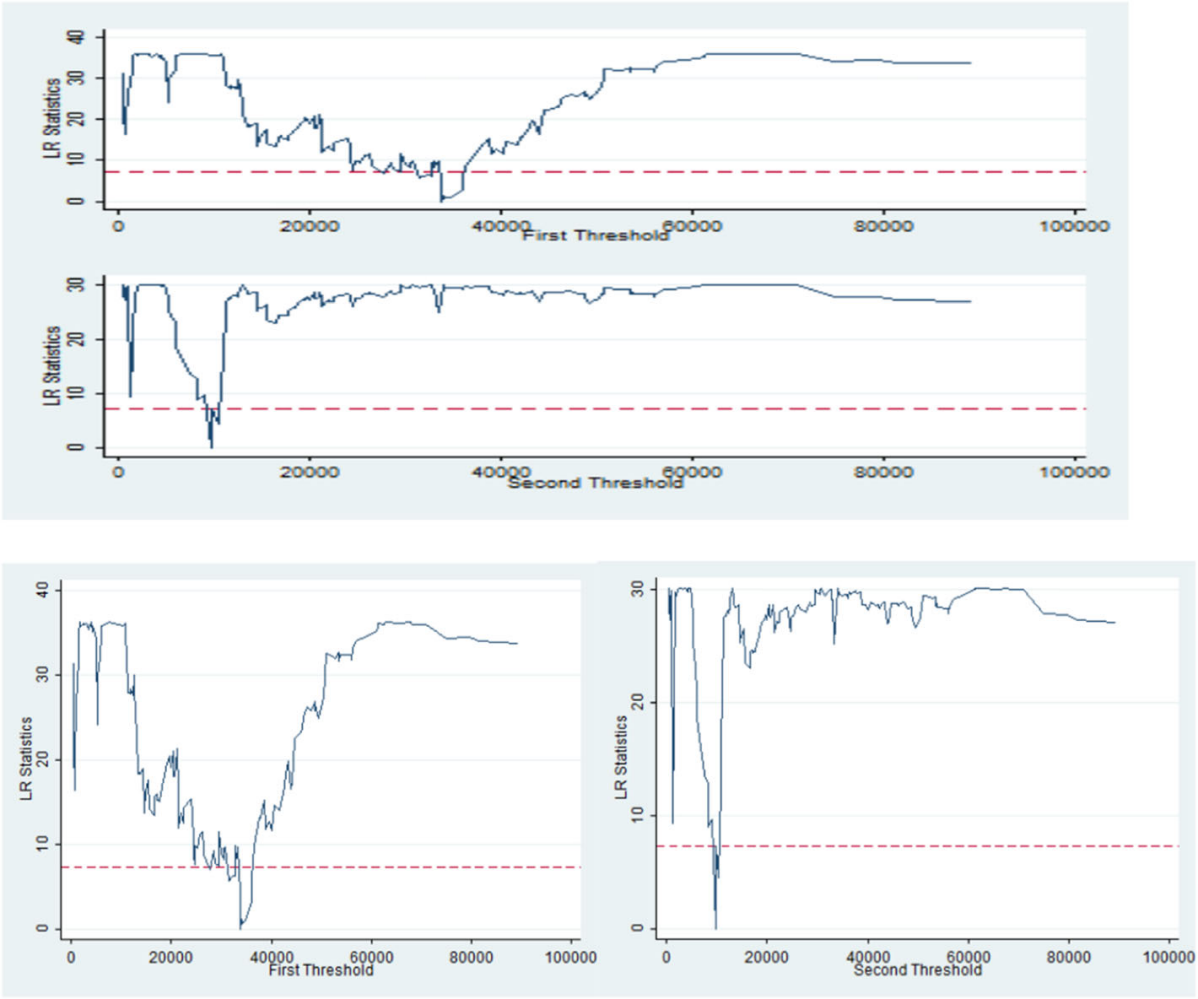
LR Statistics (Two thresholds at different levels of per capita savings have a significant impact on TB registered incidence). LR = likelihood ratio

Panel data analysis of factors related to TB registered incidence was carried out in the above-mentioned article, but has the effect of different variables on TB registered incidence been linear? In fact, the nonlinear relationship between variables is also common. Based on the linear analysis, we extended the original research from the linear framework to the nonlinear. We used the threshold regression model (TRM) to explore whether the influence of relevant factors on the registered incidence of TB under the effect of threshold variables is different in stages. We adopted panel threshold model proposed by Hansen (1999). Taking per capita savings as a threshold, we studied the influence of other independent variables on the dependent variable under the condition of different per capita savings. In order to consider the heterogeneity among different sections, an indicative function of threshold variable was introduced into the model. The specific equation is as follows y_it_ = *μ*_it_ + *β*_1_*x*_*it*_*I*(*q*_*it*_ ≤ *γ*) + *β*_2_*x*_*it*_*I*(*q*_*it*_ > *γ*) + *e*_*it*. ._ t represents time, i represents different cities, *γ* is the unknown threshold, *q*_*it*_ is the threshold variable, *e*_*it*_~*iid*(0, *δ*^2^) is the random disturbance term, and I(*) is the index function. In fact, this model is equivalent to a piecewise function model. When the threshold variable *q*_*it*_ exceeds the threshold value *γ*_*i*_, structural mutation occurs in the model. The model uses the Bootstrap method to retrieve different threshold variables (ie, various economic indicators) in order to determine whether there is a threshold effect. This method divides different cross-section individuals into different regions according to the threshold, and different intervals have different regression equation expressions. By separately estimating the corresponding coefficients, the relationship between the independent variable and the dependent variable in each area is more accurate. Firstly, the indicator per capita savings is used as the threshold, and the single threshold test shows *γ*^1^ = 9772.8086. Meanwhile, the null hypothesis that there is no threshold is rejected. The test of the two thresholds continued, showing that the second threshold value was 33,835.5391 China Yuan(CNY), which also rejected the null hypothesis with only one threshold. At this point, the first threshold value is back-checked, and fixed *γ*^2^ is back-checked for the new threshold value, and the threshold value is also 33,835.5391 CNY, indicating that there are two thresholds in this model, 9772.8086 CNY and 33,835.5391 CNY respectively. After determining the threshold value, we divided the per capita savings into three intervals: low savings <9772.8086 CNY; moderate savings 9772.8086 CNY-33835.5391 CNY; high savings > 33,835.5391CNY) and discussed on different intervals to obtain more conclusions. We can also clearly see that there are two different threshold values of the model from the Fig. 1, both of which are very significant, proving that the calculated threshold value is representative to some extent. We imported the above data into statistical software STATA14.0 and the TRM proposed by Yujun Lian (2015) was used for modeling. Based on the fixed effect model in the above-mentioned paper, some insignificant explanatory variables were deleted, and the regression results with per capita savings as the threshold variable were shown in Table 2.

**Table 2:** The Regression Results with Per Capita Savings as The Threshold Variable

Variable	RC	SD	P > |t|
Per capita GDP	0.0003178	0.000067	0.000
Urban per capita disposable income	0.0002412	0.0001623	0.139
The population density	0.125963	0.0375508	0.001
Per capita medical expenditure	−0.0253607	0.0059952	0.000
Urban engel coefficient	0.2440182	0.0862448	0.005
Rural per capita income 1	−0.0015682	0.0005788	0.007
Rural per capita income 2	−0.0028132	0.0005677	0.000
Rural per capita income 3	−0.0022253	0.0005483	0.000

Note. *TB* Tuberculosis, *RC* Regression coefficient, *SD* Standard deviation, *GDP* Gross domestic product

Rural per capita income 1, 2 and 3 means that rural per capita income has different effects on incidence under three different thresholds, so it is divided into three levels

**Fig. 2:**
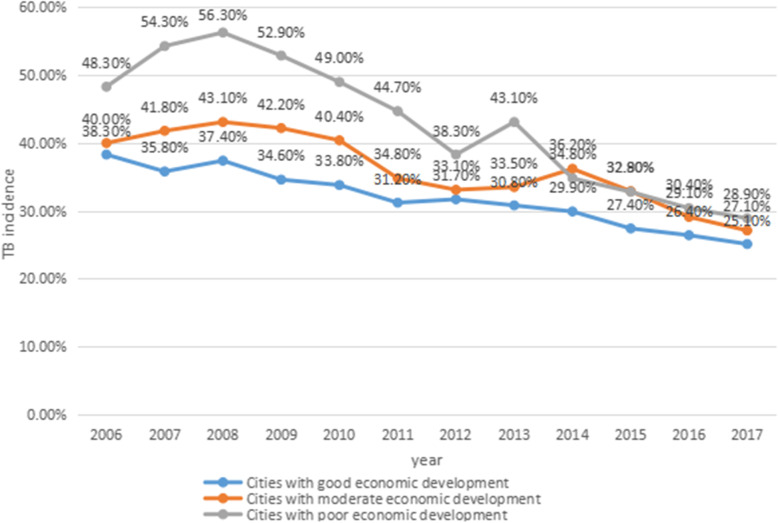
Dynamic changes in TB registered incidence in regions with different levels of economic development during 2006–2017. Note.TB = tuberculosis

To study the relationship between regional economic level and tuberculosis registered incidence, we artificially divided cities into three groups based on the GDP levels of 17 cities over the 12 years. The cities with good economic development include Jinan, Dongying, Weihai, Qingdao, Yantai and Zibo; Cities with moderate economic development include Binzhou, Laiwu, Rizhao, Weifang, Zaozhuang and Tai’an; The cities with poor economic development include Jining, Dezhou, Liaocheng, Linyi and Heze. By calculating the average of TB registered incidence in three regions, we obtained the results of dynamic changes in TB registered incidence in the three regions and the results are shown in Fig. 2.

## Results

The results of fixed effect in Table 1 show that among all the variables, per capita medical expenditure (RC, -0.0314462; SD, 0.0079305; P > |t|, 0.000) and per capita savings(RC, 0.0001924; SD, 0.0000566; P > |t|, 0.001) passed the significance test at the level of 1%. That is to say, these two variables are most closely related to the registered incidence of TB. Per capita medical expenditure is negatively correlated with TB registered incidence, and per capita savings is positively correlated with TB registered incidence.The three variables of urban per capita disposable income(RC, 0.0003896; SD, 0.0001897; P > |t|, 0.041), rural annual net income(RC, -0.0017531; SD, 0.0007222; P > |t|, 0.016) and population density(RC, 0.0983965; SD, 0.0492288; P > |t|, 0.047) passed the test at the significance level of 5%.The relationship between these variables and the registered incidence of TB is significant. Urban per capita disposable income and population density are positively correlated with TB registered incidence, while rural annual net income is negatively correlated with TB registered incidence. Per capita GDP(RC, 0.0001319; SD, 0.0000768; P > |t|, 0.088)and urban engel coefficient(RC, 0.1908406; SD, 0.0991439; P > |t|, 0.056) pass the test at the significance level of 10%.Relatively speaking, the statistical significance of these two variables is not so obvious.

Table 2 shows the impact of other indicators on TB registered incidence at different stages of per capita savings. It can be seen from Table 1 that the four economic indicators and registered incidence, such as urbanization rate, number of beds per capita, number of doctors per capita, and rural engel coefficient, have no obvious statistical significance. Therefore, they are eliminated when we perform threshold regression statistics. We take rural per capita income as an important variable for threshold regression, and the results are shown in Fig. 1. In the case of different stages of per capita savings, rural per capita income always has a significant negative impact on the registered incidence of TB (The regression coefficients are − 0.0015682, − 0.0028132 and − 0.0022253 respectively. *P* > 0.007, 0.000, 0.000 respectively.) That is to say, with the continuous increase of per capita income of rural residents, the registered incidence of TB generally decreases. However, at the same time, the impact of rural per capita income on the registered incidence of TB has a significant staged difference. From Fig. 1, we can see that when per capita savings reached the first threshold of 33,835.5391 CNY and the second threshold of 9772.8086 CNY, the impact of rural per capita income on TB registered incidence changed. At the medium savings level (9772.8086 CNY- 33835.5391CNY), rural per capita income had the greatest impact on the total registered incidence of TB.Rural per capita income had the least impact on the overall registered incidence of TB at the low savings level (less than 9772.8086 CNY).

Dynamic changes in TB registered incidence in regions with different levels of economic development during 2006–2017 are shown in Fig. 2. In cities with good economies, the peak was 38.30% in 2006.Then it fell steadily, to 25.10% by 2017. In cities with moderate economic development, the registered incidence peaked in 2008 at 43.10%.After that, there was a big fluctuation, and by 2017, it had dropped to 27.1%.In poorer cities, the registered incidence peaked in 2008 at 56.30%. Then it fell rapidly, with fluctuations, to 28.9% in 2017. The average TB registered incidence fell fastest in poorer regions, from 48.30% in 2006 to 28.90% in 2017.First of all, for more than 10 years, in order to achieve the goal of fully building a well-off society in 2020, the Chinese government has continuously increased investment in economic support and medical and health care in poor areas. Secondly, once a tuberculosis patient is diagnosed in China, he can get anti-TB drugs for free. Finally, people are engaged in routine pre-employment medical examinations, and tuberculosis patients are not allowed to enter school and enter the job. These measures are helpful for the early detection and treatment of tuberculosis patients, thereby reducing transmission and making the registered incidence rate gradually decline. But in 2007–2008, the registered incidence increased in all three regions. By 2017, the differences between the three regions were small.

## Discussion

To our knowledge, ecological studies of the TB burden have been carried out in America, Europe and Africa [5, 9–11].But such research is limited in China. This paper provides original, large population and long-term based data on the relationship between economy and TB registered incidence in the second largest province located in the eastern coast of China. Our findings show a clear ecological association between the economic development and TB registered incidence. Rural annual net income and per capita medical expenditure have significant negative effects on the registered incidence. With the increase of the above two indicators, the registered incidence shows a decreasing trend. The influence of medical expenditure on the registered incidence rate is greater than that of rural annual net income. This result corroborates the findings of a recent global analysis that social protection spending is strongly associated with lower tuberculosis case notification, incidence, and mortality rates [12]. And it also confirms the report that economic development and increased access to health care both reduce TB incidence [14]. Therefore, the government should formulate more policies to benefit the people, especially in rural areas, to promote the economic development of rural areas and alleviate poverty. More importantly, the government should increase spending on medicine and health care especially in the economically backward countryside, and actively screen for suspicious TB cases to effectively reduce the TB incidence [15, 16].

Population density, per capita savings and urban engel coefficient have significant positive effects on the registered incidence. This conclusion confirms previous research that the increased population density increases the burden of TB [17, 18].With the increase of such indicators, the registered incidence shows an increasing trend. The urban engel coefficient has the greatest impact on the TB registered incidence. Engel’s Coefficient refers to the proportion of food consumption in households. It means that the lower the household income, the larger the proportion of the household income or the total household expenditure spent on food, the larger the engel coefficient, which means the poorer the family is. Naturally, residents will spend less on health care.

Per capita GDP, urban per capita disposable income also have positive effects on the registered incidence. This result is not consistent with previous studies abroad [12]. Their data show that countries with higher GDP per capita have lower incidence and mortality of TB than others among countries with different income levels. For this result, we take into account the increase of per capita GDP and urban disposable income, which means that people’s material living conditions become better and more unhealthy lifestyles are derived, and the increasing prevalence of diabetes increases the population’s susceptibility to tuberculosis bacteria. Supporting this relationship is a wealth of evidence, with many studies showing that diabetes affects TB registered incidence and treatment outcomes [19–21]. Moreover, per capita GDP is an average indicator. When the gap between rich and poor increases, per capita GDP will no longer be representative. Therefore, we cannot exclude the interference of the above factors.

As we can see from Fig. 2, on the whole, except for small fluctuations, the registered incidence of TB in all cities showed a downward trend and it fell fastest in poorer regions. The registered incidence of TB in areas with good economic development was lower than that in areas with poor economic development, but the gap was gradually decreasing, and the registered incidence of TB in all regions was gradually stable. The following reasons might explain this phenomenon. First, areas with high economic development have better medical and educational conditions. On the one hand, the hospital’s funding for tuberculosis can be adequate. On the other hand, people’s health care awareness is relatively stronger, and the living environment is better. They will use more income for health care, medicines, nourishment, etc., so that the incidence of tuberculosis can be effectively prevented and controlled. Second, Due to economic constraints, people in poor areas will not have a strong awareness to see a doctor unless there is a major illness. Even if they are diagnosed with tuberculosis, compared with people in economically developed areas, their compliance with treatment will be worse. After the country introduced policies launched by WHO such as short-course chemotherapy, free medication, and active screening for tuberculosis before entering school, poor areas benefited more than developed areas. Therefore, while the overall registered incidence has decreased, the registered incidence in poor areas has become more significant. The studies conducted by Kui Liu and Dong D also found that economically underdeveloped areas with a high TB incidence benefit more [22, 23].Their research results also confirm our discovery and explanation.

As far as we know, this is the first study in Shandong province to assess the impact of economic development levels on TB registered incidence. Our sample data is large, including seventeen cities in Shandong province. We examined the impact of the following economic factors on TB registered incidence: per capita GDP, urban per capita disposable income and rural annual net income, population density, urbanization rate, per capita medical expenditure and the per capita savings, number of beds per capita, number of doctors per capita, rural engel coefficient, urban engel coefficient. Both linear and nonlinear patterns were explored to determine the relationship between levels of economic development and TB registered incidence. First, we used panel data regression to examine the impact of different economic factors on TB registered incidence. And then we used the TRM to extend the study from a linear framework to a nonlinear one. Through the threshold test, we selected per capita savings as a threshold variable and examined the impact of other economic factors on TB registered incidence at different levels of savings. Furthermore, we divided seventeen cities in Shandong province into three regions with different levels of economic development, and conducted a horizontal and vertical comparison to more directly explore the impact of different levels of economic development on the registered incidence of TB. Overall, increased economic input is critical, and many studies have emphasized the importance of social protection interventions to reduce the burden of TB [24, 25].

There are still some limitations in our research. First, there is a bias in the diagnosis and registration of TB patients. Most TB cases are diagnosed on the basis of clinical and radiological evidence, which may lead to some misdiagnosis and missed diagnosis. In poor areas, due to economic constraints, some people with insignificant clinical symptoms may not seek medical care, resulting in fewer TB cases registered than the true number. We have to acknowledge that some cities provided high or low registered incidence of TB. However, the extent of these inaccuracies is not known. Underestimates might arise from under-reporting or under-diagnosis of cases. Nonetheless, we believe CDC estimates are the best data available for TB registered incidence. In future research, perhaps we can reduce the bias caused by misdiagnosis and missed clinics by improving the diagnostic level of imaging doctors and encouraging people to seek medical treatment in time. Additionally, the economic development might not have an immediate effect on TB registered incidence. We considered the problem of hysteresis, but we did not know the specific length of time. Fortunately, the economic development in Shandong province has been relatively stable in recent years due to its traditional and conservative culture. In future studies, we may be able to establish a model of the delay effect between the economy and the TB registered incidence, making the study more scientific. In addition, from 2007 to 2008, the TB registered incidence increased in all areas in Shandong province and it peaked in 2008 in both poor and moderate regions. But we didn’t find a possible reason for this, so further research is needed. Moreover, if we can completely separate urban and rural areas and explore the relationship between economic indicators and TB registered incidence separately, it will be more scientific. But according to our national conditions, it is difficult to distinguish between rural and urban populations completely and accurately. As far as most provinces in China (including Shandong Province) are concerned, many people live in cities although they are registered in rural areas. However, we do not know the specific proportion. So the TB incidence in each city is the total incidence of the city’s urban and rural areas. Finally, TB is preventable and controllable both in groups and individuals, but we acknowledge that the study might not necessarily be applicable on an individual level. It can only provide certain research basis for the prevention and control of TB by relevant departments. We have not estimated the registered incidence in China. Whether these results can be used in other provinces of China and globally is controversial.

These results would strengthen the analysis and provide information for the government departments, guiding them to make economic investment decisions in TB control. For example, the government should focus on increasing economic investment in poor areas to shorten the gap between rich and poor. Relevant health departments should formulate the point-to-point support policy of the third-class hospitals for rural township hospitals, provide special training for tuberculosis diagnosis and treatment for doctors in township hospitals, and regularly carry out tuberculosis free diagnosis activities, and increase the publicity of tuberculosis-related knowledge, and reduce some tuberculosis special inspection costs. The above measures are helpful for the early detection, early diagnosis and early treatment of tuberculosis patients. A more refined measurement of economic investments on a provincial scale would be helpful to determine which programmes or policies are best at reducing TB burden. The benefits of increased economic investments go beyond TB and would be likely to affect the incidence and mortality of other communicable and non-communicable diseases, especially those with a well documented association with poverty. Our findings suggest that economic investments could contribute to a reduced TB incidence. However, further studies are needed to confirm the relationship between the level of economic development and TB incidence both in developed and developing countries.

## Conclusion

In conclusion, we found the effects of different economic factors on TB registered incidence and to what extent. Our results contribute to the understanding of which economic policies can better reduce the TB burden. In addition to developing new accurate and timely diagnostic methods, increased economic input is important for future TB control. In the economically underdeveloped areas with high prevalence in Shandong province, on the one hand, medical institutions should regularly train radiologists in various places to improve their diagnostic skills in order to reduce missed diagnosis and misdiagnosis. On the other hand, the government should increase the publicity of the basic knowledge of tuberculosis in the community to encourage the population to see a doctor in time and actively screen suspected tuberculosis cases and realize early detection and treatment by increasing medical and health investment, which will greatly reduce the morbidity and mortality of TB.

## Supplementary information

**Additional file 1.** Panal data. The panel data contains the registered incidence of tuberculosis and various economic indicators in 17 cities in Shandong Province from 2005 to 2017.

**Additional file 2.** The original data of threshold regression model. All data in the excel are the median of 12-year data (95% confidence interval).

## Data Availability

The datasets used and analysed during the current study are available from the corresponding author on reasonable request. The panel data that contains the registered incidence of tuberculosis and various economic indicators in 17 cities in Shandong Province from 2005 to 2017 is detailed in Additional file 1. The original data of threshold regression model is in Additional file 2.

## Abbreviations

TB: Tuberculosis
TRM: Threshold regression model
WHO: World Health Organization
GDP: Gross domestic product
RC: Regression coefficient
SD: Standard deviation
CDC: Chinese Center for Disease Control and Prevention
CNY: China Yuan
CPI: Consumer price index
LR: Likelihood ratio
